# Nanoenabled intracellular zinc bursting for efficacious reversal of gefitinib resistance in lung cancer

**DOI:** 10.7150/ijbs.95929

**Published:** 2024-05-19

**Authors:** Junnan Li, Yehuda G. Assaraf, Weimin Zuo, Ziqi Lin, Ka Weng Leong, Qi Zhao, Lipeng Zhu, Hang Fai Kwok

**Affiliations:** 1 Cancer Centre, Faculty of Health Sciences, University of Macau, Avenida de Universidade, Taipa, Macau SAR.; 2 Department of Biomedical Sciences, Faculty of Health Sciences, University of Macau, Avenida de Universidade, Taipa, Macau SAR.; 3 MoE Frontiers Science Center for Precision Oncology, University of Macau, Avenida de Universidade, Taipa, Macau SAR.; 4 The Fred Wyszkowski Cancer Research Laboratory, Faculty of Biology, The Technion-Israel Institute of Technology, Haifa 3200003, Israel.; 5 School of Life Sciences, Xiangya School of Medicine, Central South University, Changsha 510006, China.; 6 Key Laboratory of Study and Discovery of Small Targeted Molecules of Hunan Province, Department of Pharmacy, School of Medicine, Hunan Normal University, Changsha, Hunan, China.

**Keywords:** Lung cancer, Cancer therapy, Drug resistance, Reactive oxygen species (ROS), Zn^2+^, Zeolitic imidazolate framework-8 (ZIF-8)

## Abstract

Following the identification of specific epidermal growth factor receptor (EGFR)-activating mutations, gefitinib, one of the first-generation tyrosine kinase inhibitors (TKIs), has proven efficacious in targeting NSCLC that is driven by specific EGFR-activating mutations. However, most patients who initially respond to gefitinib, develop acquired resistance. In the current study, we devised a novel strategy to enhance the efficacy of gefitinib. We developed a simple and effective, nano-interrupter termed zeolitic imidazolate framework-8@Gefitinib@hyaluraonic nanoparticle (ZIF-8@G@HA NP). This nanoparticle was prepared by loading gefitinib onto a ZIF-8 nanoplatform followed by coating with hyaluronic acid (HA). The burst of Zn^2+^ release triggered by pH-sensitive degradation of ZIF-8@G@HA NPs was shown to enhance the efficacy of gefitinib in parental lung carcinoma HCC827 cells and overcame acquired gefitinib resistance in gefitinib drug resistant (GDR) HCC827 cells. We found that when treated with ZIF-8@G@HA NPs, Zn^2+^ acts synergistically with gefitinib via increased apoptosis in both parental and GDR HCC827 cells. Consistently, this *in vitro* activity was correlated with *in vivo* tumor growth inhibition. Interestingly, GDR cells were more sensitive to Zn^2+^ when compared with parental cells. We further found that ZIF-8 NPs overcame gefitinib resistance by triggering reactive oxygen species (ROS) generation and consequent cell cycle arrest at the G2/M phase, resulting in cancer cell apoptosis. Zn^2+^ was also found to block P-gp activity, facilitating the accumulation of gefitinib in GDR cells, thus enhancing the anti-tumor efficacy of gefitinib resulting in reversal of gefitinib resistance. Thus, this study offers a novel and promising strategy to surmount acquired gefitinib resistance via cell cycle arrest at the G2/M phase by facilitating gefitinib accumulation in GDR cells.

## Introduction

Lung cancer is one of the most life-threatening malignancies worldwide. According to the cancer mortality data, the 5-year survival rate is only 19%, second only to pancreatic cancer 1. Epidermal growth factor receptor (EGFR)-activating mutations are found in patients with non-small cell lung cancer (NSCLC), which is the major subtype of lung cancer. In this respect, various EGFR tyrosine kinase inhibitors (TKIs) have been introduced to treat NSCLC with this specific EGFR-activating mutations 2, 3. EGFR-TKIs initially achieved great success in treating NSCLC patients; for example, gefitinib has been the first-line treatment for patients with EGFR-activating mutations. However, many of the patients who were treated with gefitinib (G) subsequently developed gefitinib resistance 4-6.

Zn^2+^ is a vital cofactor for the structure and function of ~10% of the human proteome 7. Intracellular zinc levels are tightly regulated by zinc transporters, termed ZIPs and ZnTs 8, 9. Specifically, Zn^2+^ is essential for the structure and function of multiple proteins involved in DNA replication, gene expression, redox signaling, cell cycle regulation, and apoptosis. However, when excess Zn^2+^ is present intracellularly, it may exert an irreversible cytotoxic effect 7, 10. Furthermore, intracellular Zn^2+^ overload can trigger mitochondrial reactive oxygen species (ROS) production and loss of mitochondrial membrane potential (MMP), resulting in impaired ATP production 11-14. Interestingly, tumor cells are more susceptible to intracellular Zn^2+^ accumulation than normal cells since the latter have more efficient Zn^2+^ scavenging and metabolism pathways 15, 16. This difference lays the foundation for the distinct cytotoxic effect in cancer cells upon intracellular Zn^2+^ overload. Taken together, we expect that Zn^2+^-mediated ROS generation will enhance the therapeutic efficacy of gefitinib in tumor cells and may reverse gefitinib resistance. However, no report has demonstrated such a Zn^2+^-based strategy to enhance the efficacy of gefitinib and to overcome gefitinib resistance.

In this study we prepared a simple and effective nano-interrupter to overcome gefitinib resistance by inducing intracellular Zn^2+^ interference. The nano-interrupter was constructed by loading gefitinib into a zeolitic imidazolate framework-8 (ZIF-8) nanoplatform and then coating it with hyaluronic acid (HA), termed ZIF-8@Gefitinib@HA nanoparticle (ZIF-8@G@HA NPs). The NPs were decorated with HA which conferred an ability to bind to the surface HA receptor CD-44 and undergo internalization into NSCLC cells which frequently express this stem cell-like marker 17, 18. Moreover, HA decoration also provided the nano-interrupter with better stability and biocompatibility. ZIF-8@G@HA NPs were found to effectively encapsulate gefitinib, hence overcoming the low aqueous solubility of gefitinib in clinical applications. Benefiting from the pH-triggered structural collapse, ZIF-8 can trigger the release of Zn^2+^ in the acidic tumor microenvironment as well as in lysosomes upon receptor-mediated endocytosis. Our results show that ZIF-8@Gefitinib@HA could significantly enhance gefitinib efficacy in parental HCC827 cells and it could also reverse gefitinib resistance in GDR cells. Although intracellular Zn^2+^ accumulation in tumor cells can promote ROS generation in both tumor cell lines, gefitinib-resistant HCC827 cells were more sensitive to the release of Zn^2+^. Moreover, our results show that Zn^2+^ could also inhibit P-glycoprotein (P-gp) synthesis and induce G2/M cell cycle arrest in GDR cells but not in parental HCC827 cells. P-gp, one of the major ATP-binding cassette (ABC) multidrug efflux transporters has been reported to play a crucial role in conferring multidrug resistance upon tumor cells 19, 20. These efflux transporter proteins were suggested to markedly reduce therapeutic efficacy by acting as drug efflux pumps in cancer cells 21. Thus, upon major intracellular Zn^2+^ accumulation, less gefitinib would be pumped out and Zn^2+^ interference-induced cell cycle arrest could also trigger potent antitumor activity in GDR cells. Consequently, this nano-interrupter could achieve a superior synergistic activity and lead to effective suppression of tumor cell/tissue growth. This effective "Zn^2+^ interference" mechanism has potential for clinical application.

## Materials and Methods

### Reagents

Gefitinib (Cat#: 8219) was purchased from ApexBio Technology (Houston, TX, USA). Zinc nitrate hexahydrate (96482), 2-methylimidazole (M50850), N-acetyl cysteine (A9165) and EDTA (E6758) were obtained from Sigma-Aldrich. Hyaluronic acid (HA) was purchased from Bloomage Biotechnology Corporation Limited (Baiyun District, Guangzhou). Peroxidase-conjugated AffiniPure goat anti-mouse (SA00001-1) and peroxidase-conjugated AffiniPure goat anti-rabbit (SA00001-2) were purchased from Proteintech Group (Rosemont, PA, USA). All other chemicals were acquired from Sigma-Aldrich (St Louis, MO, USA).

### Preparations of ZIF-8 and ZIF-8@G@HA nano-interrupters

The preparation methods for ZIF-8 and ZIF-8@G were described in a previous report 22. In detail, a stock solution of gefitinib (40 mg/mL) was prepared in methanol. First, 20 µl of zinc nitrate hexahydrate (1g/ml) was dissolved in 600 µl of methanol after which 25 µL of gefitinib stock solution was added to the zinc nitrate hexahydrate solution. The mixture was stirred for 5 min at room temperature, and 600 µL of 2-methylimidazole solution (55 µg/ml) was added dropwise. The solution was stirred again for 15 min. The product was collected by centrifugation (13,000 x g for 10 min). Solid nanoparticles (ZIF@G NPs) were obtained at room temperature by vacuum drying. ZIF-8 was prepared by adding methanol instead of the gefitinib stock solution to a zinc nitrate hexahydrate solution. HA-coated ZIF-8@HA/ZIF-8@G@HA was prepared by adding 1 mL of a 5 mg/mL HA aqueous solution to the above products. The solution was gently stirred for 48 h at room temperature, following which the samples were washed with deionized water three times to remove free HA. The resulting ZIF-8@HA/ZIF-8@G@HA NPs were dried in vacuum. The drug loading efficiency (DLE) of ZIF-8@G@HA was calculated to be 60.8%, according to the previously reported equation:

DLE (%) = (weight of loaded drug)/(total amount of drug in feed) ×100%

### Characterization of nanoparticles

Morphology and structure of the nanoparticles were determined using transmission electron microscopy (TEM) with a FEI Tecnai G2 S-Twin containing a field emission gun operating at 200 kV. Size distributions and zeta potentials of the nanoparticles were investigated using a Malvern instrument Zetasizer Nano system at room temperature.

### *In vitro* Zn^2+^ release from ZIF-8@G@HA

ZIF-8@HA/ZIF-8@G@HA nanoparticles were sufficiently dissolved in phosphate bufferred saline (PBS) at pH values of 7.2 and 5.4 for different time points. Subsequently, the mixture was centrifuged at 13,000xg for 20 min, and the supernatant was collected to detect Zn^2+^ concentrations by inductively coupled plasma mass spectrometry (ICP-MS).

### Disintegration of ZIF-8@G@HA *in vitro*

ZIF-8@G@HA NPs were processed as described above and sufficiently dissolved in PB buffer (pH 7.2, pH 5.4), respectively. Subsequently, the mixture was centrifuged at 13000 x g for 20 min. The precipitate was collected for TEM analysis.

### Establishment of EGFR-TKIs resistant cells

HCC827 cells were purchased from the Shanghai Institute for Biological Sciences (Chinese Academy of Sciences). HCC827 cells were cultured in RPMI-1640 medium containing 10% fetal bovine serum (Gibco™, Grand Island, New York). Gefitinib-resistant HCC827 cells (GDR) were established by culturing parental cells in stepwise increasing concentrations of gefitinib (100 nM-10 µM). Growth medium and drugs were refreshed twice weekly during establishment of drug resistance.

### mRNA extraction and RNA-sequencing

Total RNA was extracted using an RNeasy Kit (74136, Qiagen, Germany), and cDNA libraries were constructed with NEBNext® Ultra™ Directional RNA Library Prep Kit for Illumina® (NEB #E7760, New England Biolabs, Ipswich, MA, USA). The cDNA libraries were sequenced using an Illumina Hi-Seq (Illumina, San Diego, CA). Raw RNA-sequencing data were subjected to FastQC. Quality confirmed RNA-sequencing reads from each library were aligned and mapped with TopHat 2.1.1 (Center for Computational Biology at Johns Hopkins University). Expression quantification was defined by fragments per kb of transcript per million mapped reads via Cufflinks 2.2.1 (Trapnell-Lab, Github) after mapping. Filtered gene sets were identified as upregulated or downregulated by having more than a 1.5-fold change in paired samples. For the functional annotation of the transcriptome profiles, genes identified to be differentially expressed were subject to GO analysis using the web-based tool, DAVID v6.8 (The Database for Annotation, Visualization, and Integrated Discovery, National Institute of Allergy and Infectious Diseases, NIH).

### Construction and analysis of Protein-Protein Interaction (PPI) network

After collecting the genes that were introduced in the articles, it is crucial to identify the principal proteins and biochemical pathways involved. Towards this end, constructing a network of protein interactions is necessary. The STRING is a tool that helps predict the interactions and function of genes upon reconstruction of networks. The STRING database, specifically version 10.5 (available at https://string-db.org), was employed to build a Protein-Protein Interaction (PPI) network from the list of genes. This network illustrates connections between protein pairs based on their similar biological functions.

### MTT assay

MTT assay (MTT, Sigma, Cat#: M2128) was performed to determine the efficacy of the different treatments. Briefly, GDR cells were seeded in a 96-well plate (5,000 cells/well) and incubated for 24 h. After exposure to drugs for 24 h, 10 μL of an MTT solution were added to each well, and the cells were incubated for another 2-4 h. The supernatant was then discarded, and 100 μL of dimethyl sulfoxide (DMSO) was added to dissolve the formazan crystals. Plates were then measured at 570 nm using Thermo Scientific Microplate Reader (Multiskan Spectrum) for optical density (OD) determination.

### JC-1 staining assay to evaluate mitochondrial membrane potential (MMP)

GDR cells were seeded in a 6-well plate (2x10^5^ cells/well) and incubated for 24 h. After exposure to the different treatments for 24 h, mitochondrial membrane potential** (**MMP) was measured using the MMP assay kit with JC-1 staining (C2006, Beyotime Biotechnology, China) according to the manufacturer's instructions. Cells were washed twice with PBS after different treatments and then incubated with JC-1 at 37°C for 30 min. After incubation, cells were washed twice with PBS, and the EVOS M7000 Imaging system observed cellular fluorescence.

### Reactive oxygen species (ROS) detection

Cells were loaded with DCFH-DA (10 μM) in RPMI-1640 without FBS for 30 min at 37^o^C to detect the production of ROS after different treatments. After washing three times with PBS, cells were observed under a fluorescence microscope (EVOS M7000 Imaging system).

### Analysis of intracellular zinc ion release

Cells were stained with 5 μM FluoZinTM-3 acetoxymethyl (AM) at 37^o^C for 30 min after treatments. Cells were then washed in sterile PBS, visualized by fluorescence microscopy, and analyzed using a BD Accuri C6 flow cytometer (BD Biosciences, San Jose, CA).

### Cell cycle assay

Cells were seeded in a 6-well plate (2x10^5^ cells/well) and incubated for 24 h. Cells were then collected and washed with PBS after different treatments. Cells were then fixed with cold 70% ethanol overnight at -20 °C. Cells were then resuspended and incubated for 30 min at 37°C in 300 µL of 200 µg/ml DNase-free RNase A to remove RNA (Thermo Fisher, 12091021). After washing with PBS, cells were stained with 500 µl of 50 μg/ml PI (Sigma-Aldrich, P-4170) containing 0.1% Triton X-100 (Sigma-Aldrich, X100) for 15 min at room temperature. DNA content was determined for cell cycle analysis using a BD Accuri C6 flow cytometer (BD Biosciences, San Jose, CA). FlowJo software (Tree Star, Inc.) was used to quantify cell cycle distribution.

### Live/dead cell staining assessment

Cells were seeded in a 12-well plate (10^5^ cells/well) and incubated for 24 h. After different treatments, cells were subsequently stained with Calcein AM/PI for 15 min and then imaged using an inverted fluorescence microscope (EVOS M7000 Imaging system).

### Cell apoptosis assays

Cells were seeded in a 12-well plate (10^5^ cells/well) and incubated for 24 h after which cells were collected and washed with PBS after different treatments for 24 h. Cells were then suspended and stained with 0.1 mL Annexin V binding buffer containing 1 μg /mL PI (Sigma-Aldrich, P-4170) and Annexin V-FITC (ThermoFisher, A13201) for 15 min at room temperature. After incubation, 400 µl annexin-binding buffer was added to cells to stop staining. Cell apoptosis was detected using a BD Accuri C6 flow cytometer. FlowJo software (Tree Star, Inc.) was used to quantify cell cycle distribution.

### Xenograft tumor mouse models

All animal procedures were approved by the Animal Research Ethics Committee of the University of Macau and were performed according to the ARRIVE guidelines. All animals were maintained at the Specific Pathogen Free (SPF) Animal Facility, University of Macau. Four-week-old female nude mice (The Jackson Laboratory, Bar Harbor, ME) were used for tumor xenografts. Mice were housed in a fully climate-controlled room with constant temperature and humidity and free access to food and water. GDR cells (1×10^7^ cells/mouse) were suspended in Matrigel and implanted subcutaneously into nude mice. When tumors became palpable, mice were divided into five groups including: the PBS group; Gefitinib group; ZIF-8@HA; Gefitinib+ ZIF-8@HA and the ZIF-8@G@HAgroup. Since gefitinib is insoluble in water and PBS, thus the administration routes of Gefitinib and NPs are different. The administration routes are as follows: (1) PBS (via oral administration), (2) Gefitinib (25 mg/kg, via oral administration), (3) ZIF-8@HA (20mg/kg, via intravenous injection), (4) Gefitinib (25 mg/kg, via oral administration) + ZIF-8@HA (20mg/kg, via intravenous injection) and (5) ZIF-8@G@HA (20mg/kg, via intravenous injection). Tumor size was measured periodically by calipers, and the tumor volume (mm^3^) was determined by the ellipsoid formula (volume = L × W^2^ /2). Mouse body weight was measured regularly to assess drug toxicity.

### H&E and TUNEL staining

After mice had been sacrificed, tumors were extracted for a histological study. Tumors were fixed in 4% paraformaldehyde, and slides were prepared for further studies. For H&E staining, slides were stained with both Mayer's H&E studies. For apoptosis analysis of tumor tissues, the TUNEL Apoptosis Assay Kit (Beyotime, C1088) was used to detect apoptosis. Five fields from each slide from three mice in each group were chosen and analyzed. The percentage of apoptosis was calculated by Image Pro Plus 6.0 using the mean density of Integrated optical density (IOD).

### Western blot analysis

Proteins were resolved on 12% sodium dodecyl sulfate polyacrylamide gel electrophoresis (SDS-PAGE) and then transferred to a nitrocellulose membrane. Membranes were blocked in 5% skim milk in PBS for 1 h at room temperature, incubated with primary antibody overnight at 4°C and subsequently with its respective secondary antibody at room temperature for 1 h. Bands were quantified using Odyssey CLx Imaging System (LI-COR).

### Statistical analysis

All data were presented as means ± standard deviation (SD). A Student's t-test or one-way analysis of variance (ANOVA) on GraphPad Prism5 showed statistical significance between the two groups. P < 0.05 and P < 0.01 values were considered statistically significant.

## Results

### Synthesis and Characterization of ZIF-8@G@HA Nano-interrupters

**Figure 1:**
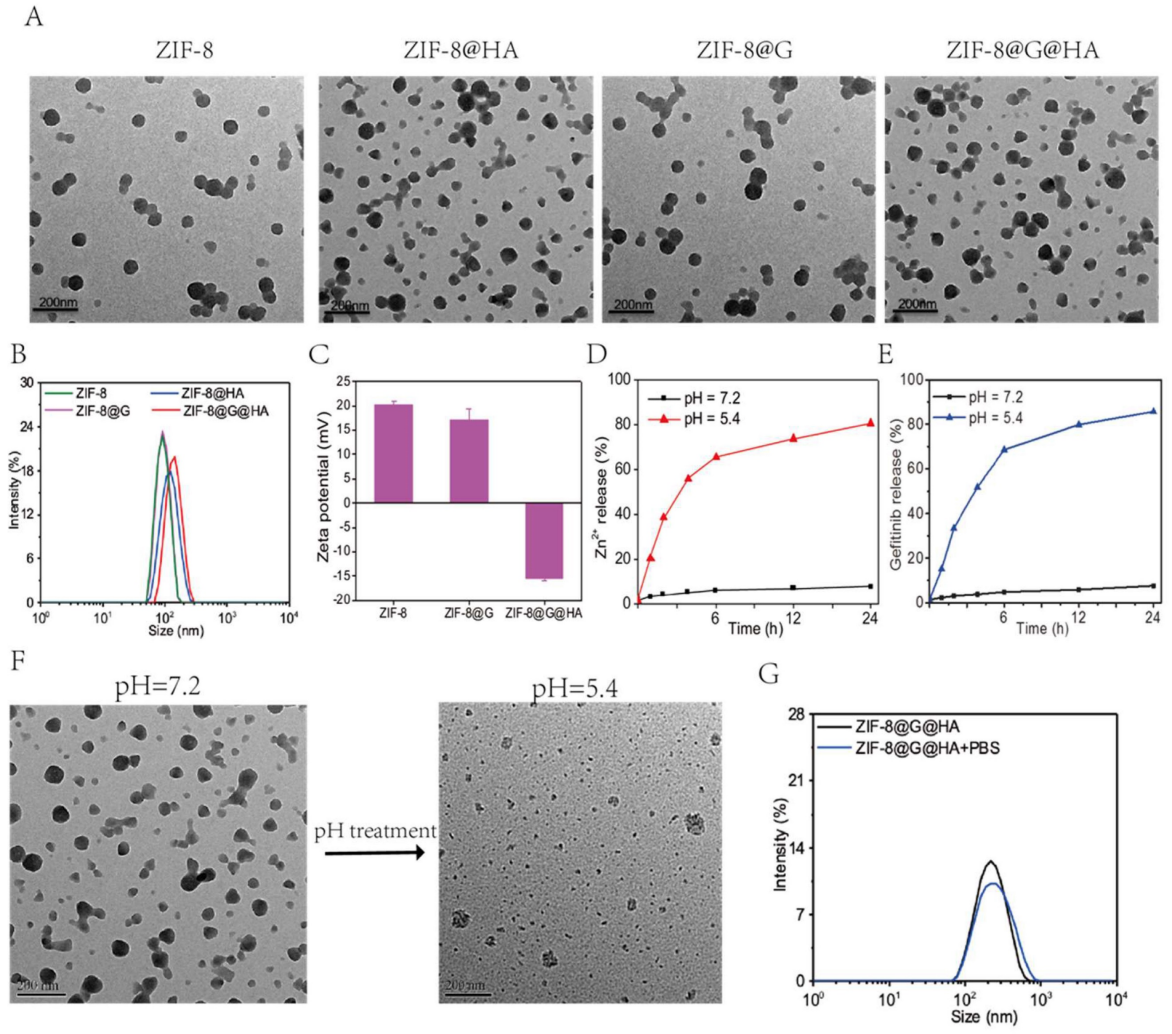
** Preparation and characterization of ZIF-8@G@HA.** (A) Transmission electron microscopy (TEM) image of ZIF-8, ZIF-8@HA, ZIF-8@G, and ZIF-8@G@HA NPs. (B) Average particle sizes of ZIF-8, ZIF-8@HA, ZIF-8@G, and ZIF-8@G@HA. (C) Zeta potential profiles of ZIF-8, ZIF-8@G, and ZIF-8@G@HA (D) The pH-responsive release of Zn^2+^ from ZIF-8@G@HA in pH 5.4, and pH 7.2. (E) The pH-responsive release of gefitinib from ZIF-8@G@HA in pH 5.4, and pH 7.2. (F) Representative TEM images of ZIF-8@G@HA at pH 7.4 and pH 5.4. (G) Representative size distribution of ZIF-8@G@HA in water and physiological buffer (phosphate-buffered saline [PBS]) (scale = 100 nm).

In this study, ZIF-8 NPs were synthesized using a simple one-pot process, and gefitinib was loaded into ZIF-8 NPs to overcome the low aqueous solubility of gefitinib in clinical applications. 23, 24. To further enhance the stability and biocompatibility of these NPs as well as equip them with an ability to bind to CD-44 and undergo internalization into CD-44 expressing tumor cells, HA decoration was introduced as a coating for of ZIF-8@G NPs to facilitate the construction of the multifunctional ZIF-8@G@HA nano-interrupters (Schematic 1). As evident from the transmission electron microscopy (TEM) images depicted in Fig. 1A, ZIF-8, ZIF-8@HA, ZIF-8@G, and ZIF-8@G@HA exhibit similar sizes and morphologies less than 150 nm with narrow size distributions. After being decorated with HA, the HA supports the nanomaterials on the surface like a membrane structure. Mean particle sizes of ZIF-8, ZIF-8@HA, ZIF-8@G, and ZIF-8@G@HA were about 89, 122, 92, and 142 nm, respectively, as indicated by dynamic light scattering (DLS) measurements (Fig. 1B). ZIF-8 and ZIF-8@G displayed similar average sizes, suggesting no obvious size change after embedding gefitinib into the ZIF-8 nanoplatform, whereas after coating with HA, the size of ZIF-8@G@HA increased slightly. Concurrently, the zeta potentials of the NPs were determined using DLS to identify the surface property changes. As shown in Fig. 1C, ZIF-8@G exhibited lower surface zeta potential (17.21 mV) than ZIF-8 (20.27 mV). Additionally, when comparing the zeta potential of ZIF-8@G@HA with that of ZIF-8@G, the value was found to decrease substantially from 17.21 to -15.31 mV due to successful HA-induced surface modifications (Fig. 1C). Zn^2+^ and gefitinib release from ZIF-8@G@HA NPs at different pH values (7.2 and 5.4) was investigated (Fig. 1D, E). Zn^2+^ and gefitinib were efficiently released from ZIF-8 NPs at an acidic pH of 5.4, and the release rate was faster than that at a physiological pH of 7.2. These results indicate that the release of Zn^2+^ and gefitinib from ZIF-8@G@HA NPs occurs in an acidic pH-dependent manner. Concurrently, the pH responsible for drug release behavior of Zn^2+^ from ZIF-8@G@HA NPs is depicted in Fig. 1F. TEM images allowed us to observe a complete structural collapse of ZIF-8@G@HA in an acidic pH. Due to the pH-responsive capability, the structure completely collapsed at an acidic environment. Besides, no obvious size change was observed for ZIF-8@G@HA NPs in different solutions (water or phosphate-buffered saline [PBS]), demonstrating that the surface modification of HA ensured the stability of ZIF-8@G@HA NPs (Fig. 1G).

### Cellular uptake and drug delivery properties of ZIF-8@G@HA NPs in parental HCC827 cells

**Figure 2:**
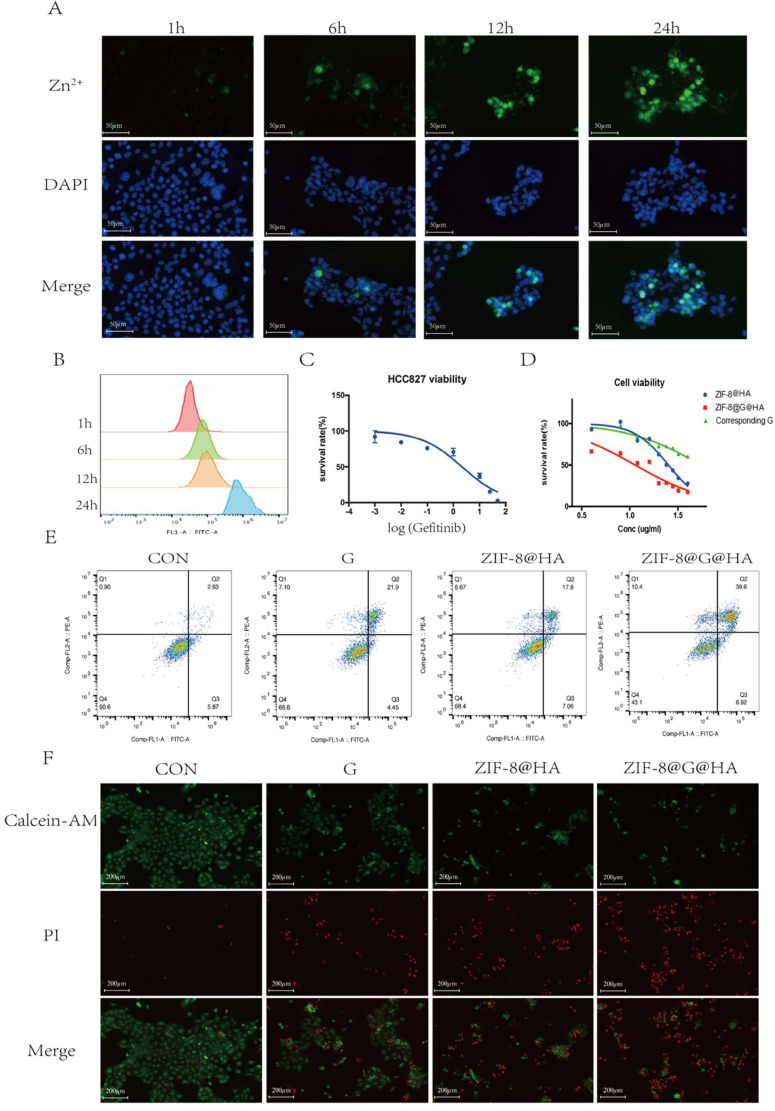
** Efficacy of ZIF-8@G@HA NPs in parental HCC827 cells.** (A) Fluorescence microscopy images of Zn^2+^ release in HCC827 cells after treatment with ZIF-8@G@HA NPs for 1,6,12 and 24 h (scale bar = 50 μm). (B) The corresponding flow cytometric analysis of Zn^2+^ release in HCC827 cells after treatment with ZIF-8@G@HA NPs for 1,6,12 and 24 h. (C) Detection of HCC827 cell viability upon gefitinib treatment for 24h using the tetrazolium MTT assay. (D) Detection of HCC827 cell viability upon treatment with ZIF-8@HA/ZIF-8@G@HA NPs and the corresponding gefitinib within ZIF-8@G@HA NPs for 24h using the MTT assay. (E) HCC827 cell apoptosis analysis after treatments of gefitinib/ZIF-8@HA/ZIF-8@G@HA for 24h. (F) Live and dead HCC827 cell staining images after treatments of gefitinib/ZIF-8@HA/ZIF-8@G@HA for 24h (scale bar = 200 μm).

To investigate the effects of endocytosis and intracellular drug release of ZIF-8@G@HA NPs, cellular uptake into parental HCC827 cells was investigated using microscopy and flow cytometry. Cells were incubated with ZIF-8@G@HA NPs for 1, 6, 12, and 24 h and the intracellular accumulation of Zn^2+^ was determined by staining by FluoZinTM-3 acetoxymethyl, a viable fluorescent indicator of Zn^2+^. As shown in Fig. 1A, cells displayed a very weak green fluorescence signals both after 1 and 6 h of incubation, suggesting that only a small amount of Zn^2+^ was released into the cytoplasm. However, after 12 h of incubation, the intracellular green fluorescence signal increased remarkably. Furthermore, an even stronger and more diffuse signal could be observed after the cells were incubated with the 8@G@HA NPs for 24 h. These findings suggest that the amount of cellular uptake of the zinc cations released from the endocytosed nanoparticles increased over time. Quantitative flow cytometry analysis of intracellular Zn^2+^ for samples obtained at 1, 6, 12 and 24 h of incubation showed consistent results (Fig. 2B). The increasing fluorescence signal demonstrates that the zinc capsule of the nanoparticles could be broken down intracellularly into zinc cations through a time-dependent manner, resulting in a marked intracellular accumulation of Zn^2+^. Together, the Zn^2+^ nanoplatform emerges as a promising delivery carrier of gefitinib.

### ZIF-8@G@HA enhanced gefitinib-induced cytotoxicity in non-resistant HCC827 cells *in vitro*

We next explored the cytotoxic activity of ZIF-8@G@HA NPs in parental HCC827 cells. We first determined the IC_50_ value of gefitinib in parental cells using an MTT assay in which the IC_50_ value was 0.89 **±** 0.25 μM (Fig. 2C). We then conducted a cytotoxicity assay to compare the effects of zinc-based nanoplatforms with and without incorporation of gefitinib on parental cells. We evaluated the 24-hour survival of cells treated with different concentrations of these nanoparticles. As depicted in Fig. 2D, gefitinib-loaded nanoparticles, ZIF-8@G@HA, exhibited a stronger inhibitory effect on cell viability compared to the unloaded NPs. Additionally, we specifically determined the cell viability associated with the corresponding concentration of gefitinib within the ZIF-8@G@HA nanoparticles, as shown in Fig. 2D. The results confirmed that the presence of gefitinib within the ZIF-8@G@HA nanoparticles could also inhibit cell viability of HCC827 cells. This observation further supports the potentiation of gefitinib's therapeutic efficacy when incorporated into the ZIF-8@G@HA nanoparticles. Furthermore, the effects of empty ZIF-8@HA NPs in combination with gefitinib were determined using the combination index (CI) described by Chou and Talalay 25. CI values less than 1 indicate synergism, whereas CI values larger than 1 indicate antagonism, a value of 1 indicates a simple addition. The CI value obtained was 0.70±0.07, which indicates a synergistic effect of ZIF-8@G@HA.

We then assessed cell apoptosis using an Annexin-V/propidium iodide double-staining assay. Similar cell apoptosis levels were observed in parental cells that had received only gefitinib or empty ZIF-8 treatment; however, significantly higher apoptosis levels were obtained when HCC827 cells were exposed to ZIF-8@G@HA (Fig. 2E). Hence, dead and live cells were followed by staining with propidium iodide (PI, red fluorescence) and calcein acetoxymethyl ester (calcein-AM, green fluorescence), respectively (Fig. 2F). Consistent with the apoptosis obtained from the Annexin-V/PI double-staining assay, most dead cells could be observed after incubation with ZIF-8@G@HA. Taken together, the zinc nanoplatform appears to enhance gefitinib efficacy in parental HCC827 cells and exerts a synergistic activity in conjunction with gefitinib in tumor cell kill.

### ZIF-8@G@HA enhance gefitinib-induced cytotoxicity in parental HCC827 cells *in vivo*

**Figure 3:**
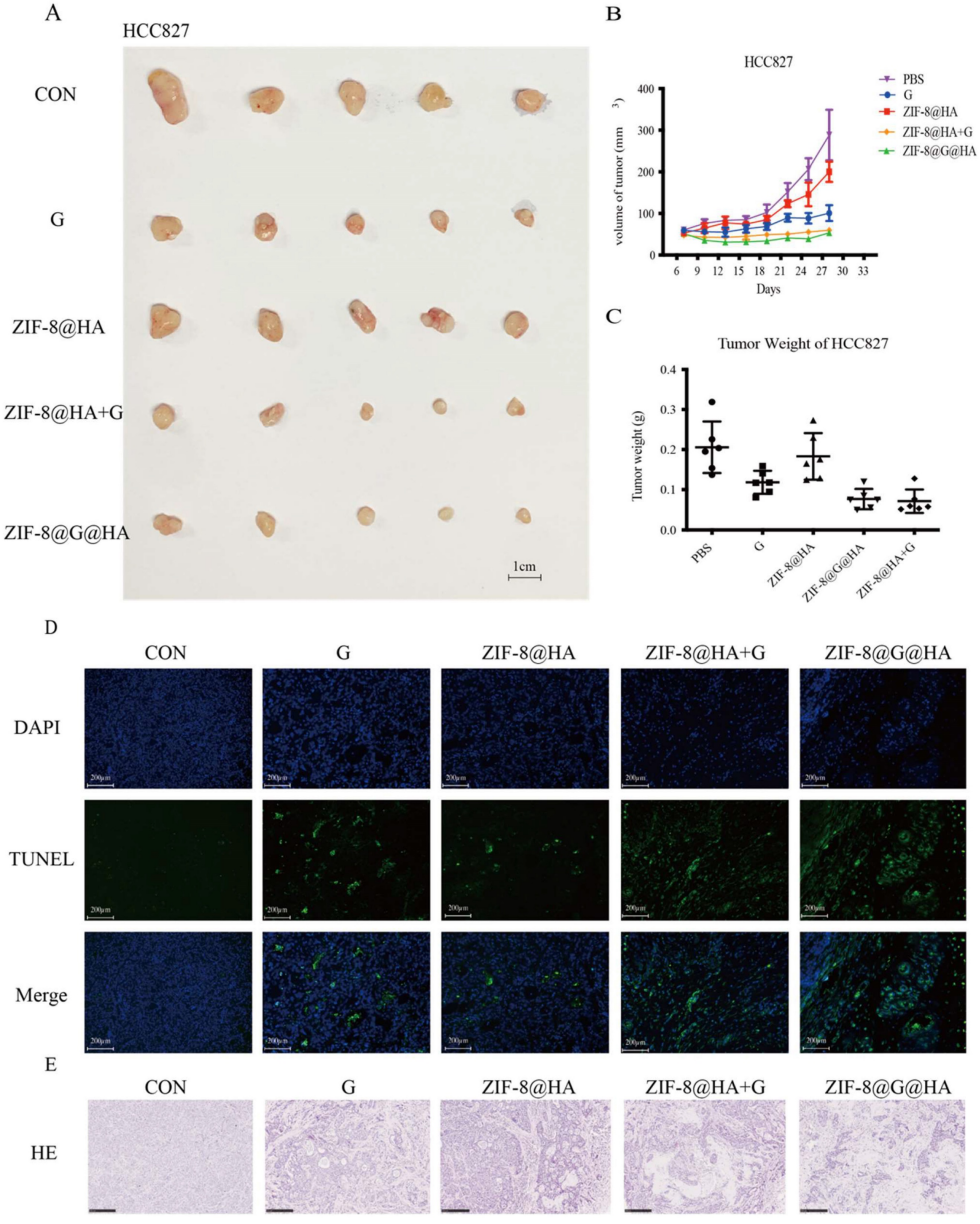
** Efficacy of the ZIF-8@G@HA NPs in HCC827 xenograft mice.** (A-C) Analysis of tumor progression in terms of tumor size, tumor volume, and tumor weight after tumor excision from HCC827 xenograft mice that received different treatments for three weeks: (1) PBS (via oral administration), (2) Gefitinib (25 mg/kg, via oral administration), (3) ZIF-8@HA (20mg/kg, via intravenous injection), (4) Gefitinib (25 mg/kg, via oral administration) + ZIF-8@HA (20mg/kg, via intravenous injection) and (5) ZIF-8@G@HA (20mg/kg, via intravenous injection). (D) Tumor cell apoptosis in the excised HCC827 tumors. Scale bar= 200 μm. (E) H&E staining of the paraffin-embedded sections of the HCC827 tumor tissues (scale bar = 250 μm).

To explore the activity of ZIF-8@G@HA on parental HCC827 tumors *in vivo*, we treated HCC827 xenograft-bearing mice with oral PBS, oral gefitinib, i.v. ZIF-8@HA, i.v. ZIF-8@G@HA, or a combination of oral gefitinib and i.v. ZIF-8@HA. As shown in Fig. 3A-C, treatment with ZIF-8 NPs alone resulted in a slight reduction in tumor size of HCC827 xenograft-bearing mice, whereas gefitinib treatment alone significantly inhibited tumor growth. Our findings further revealed that the combination treatment of empty ZIF-8 NPs and gefitinib resulted in an increased inhibition of tumor growth when compared with the single agent treatments. Moreover, ZIF-8@G NPs which were loaded with gefitinib displayed a similar efficacy when compared with the combination treatment of ZIF-8 NPs and gefitinib. These findings suggest that the combination of ZIF-8 NPs and gefitinib, as well as the use of ZIF-8@G NPs, bear potential therapeutic applications for inhibition of tumor growth in HCC827 xenograft mouse models. Most importantly, one should note that the dose of gefitinib embedded in ZIF-8@G is significantly lower at a dose of 0.6mg/kg compared to the oral treatment with gefitinib at a 42-fold higher dose of 25 mg/kg. Hence, these results indicate that by incorporating gefitinib into ZIF-8 NPs, the resultant ZIF-8@G NPs offer a novel and simple therapeutic approach, to enhance tumor growth inhibition, thus maximizing their cytotoxic efficacy. This innovative two-in-one system provides the advantages of targeted drug delivery. The combined intervention of Zn^2+^ release and gefitinib delivery through the ZIF-8@G@HA platform appears to synergistically enhance the therapeutic efficacy, resulting in significantly improved tumor growth inhibition (Fig. 2A-C). We consistently observed a significant increase in tumor cell apoptosis in the xenografted mice upon treatment with the combination of ZIF-8@G instead of the other single agent treatments (Fig. 3D). In addition, H&E staining revealed more and larger cystic spaces, indicating increased necrosis, in mice treated with the combination of ZIF-8@G compared to those that received the monotherapy treatments (Fig. 3E). Taken together, these findings further emphasize the potential of the ZIF-8@G strategy as an efficacious nanomedicine approach for lung cancer treatment.

### The *in vitro* and *in vivo* effects of the ZIF-8@G@HA on gefitinib-resistant HCC827 cells (GDR cell line)

**Figure 4:**
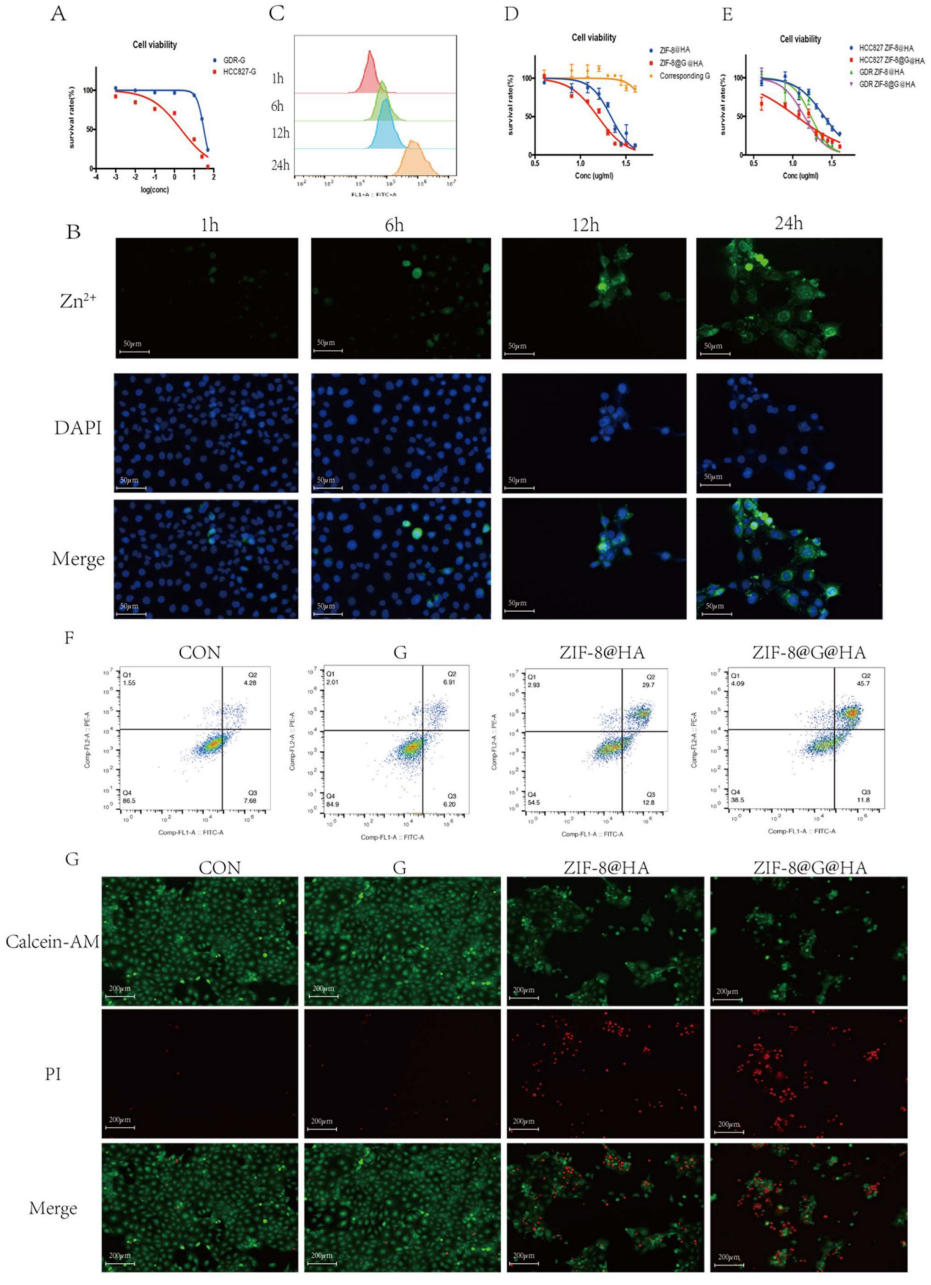
** Efficacy of the ZIF-8@G@HA NPs in GDR cells.** (A) Determination of cell viability after gefitinib treatment of both parental HCC827 cells and GDR cells using the tetrazolium MTT assay. (B) Fluorescence microscopy images of Zn^2+^ release in GDR cells after treatment with ZIF-8@G@HA NPs for 1,6,12 and 24 h (scale bar = 50μm). (C) The corresponding flow cytometric analysis of Zn^2+^ release in GDR cells after treatment with ZIF-8@G@HA NPs for 1,6,12 and 24 h. (D) Determination of cell viability after treatment with ZIF-8@HA/ZIF-8@G@HA NPs and the corresponding gefitinib within ZIF-8@G@HA NPs for 24h in GDR cells using the MTT assay. (E) Comparison of the viability of parental HCC827 cells and GDR cells after different treatments. (F) Cell apoptosis of GDR cells after different treatments. (G) Live and dead staining images of GDR cells with different treatments after culturing for 24 h (scale bar = 200 μm).

To determine whether ZIF-8@G@HA could restore the efficacy of gefitinib in NSCLC cells with acquired gefitinib resistance, we established a gefitinib drug-resistant (GDR) HCC827 cell line by growing the cells in gradually increasing concentrations of gefitinib. As shown in Fig. 4A, GDR cells displayed resistance to gefitinib. The cellular uptake of ZIF-8@HA NPs was followed in GDR cells using staining with FluoZinTM-3 acetoxymethyl staining, a viable fluorescent dye which detects Zn^2+^. The results showed that the fluorescence signals were increased significantly in a time-dependent manner, indicating that the amount of cellular uptake of Zn^2+^ released from the NPs increased as a function of time (Fig. 4B-C). We then studied the cytotoxicity of ZIF-8@G@HA in GDR cells. While treatment with gefitinib alone at the corresponding concentration of gefitinib within the ZIF-8@G@HA NPs could no longer induce cytotoxicity in GDR cells, a significant increase in cell apoptosis was observed in ZIF-8@HA-treated GDR cells, and the loading of gefitinib within ZIF-8@G@HA NPs significantly enhanced its inhibitory activity as reflected in the decreased viability of GDR tumor cells (Fig. 4D). Moreover, the MTT assay results suggest that GDR cells may be more sensitive to the release of Zn^2+^ when compared with their parental HCC827 cells, suggesting that Zn^2+^ has the potential to reverse gefitinib resistance in GDR cells (Fig. 4E). This observation indicates that the presence of Zn^2+^ within the nanoparticle formulation can restore gefitinib sensitivity in GDR cells.

**Figure 5:**
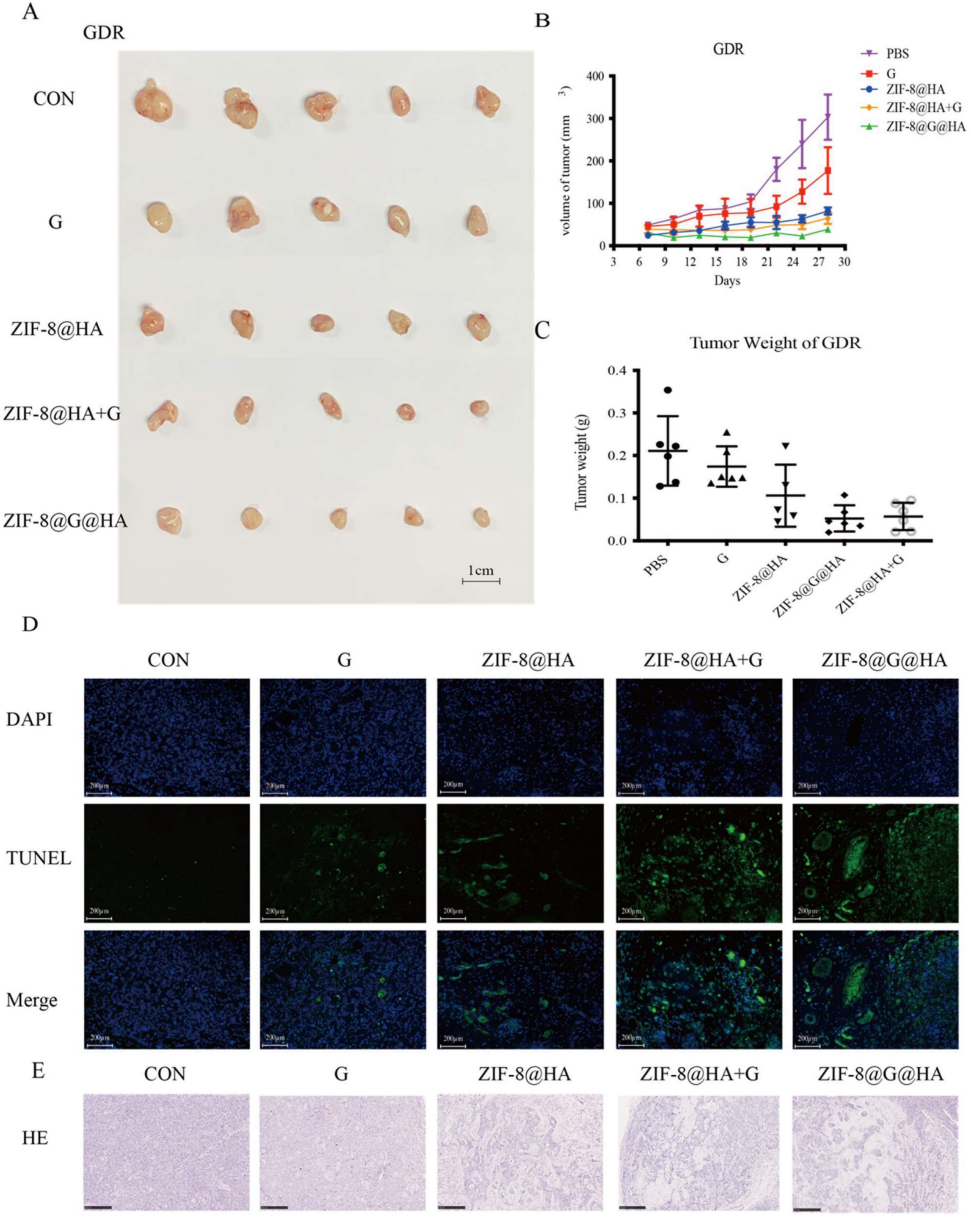
** Efficacy of the ZIF-8@G@HA NPs in mice bearing GDR xenografts.** (A-C) Analysis of tumor progression in terms of the tumor size, tumor volume, and tumor weight of the tumors excised from the GDR xenograft mice that received different treatments for three weeks: (1) PBS (via oral administration), (2) Gefitinib (25 mg/kg, via oral administration), (3) ZIF-8@HA (20mg/kg, via intravenous injection), (4) Gefitinib (25 mg/kg, via oral administration) + ZIF-8@HA (20mg/kg, via intravenous injection) and (5) ZIF-8@G@HA (20mg/kg, via intravenous injection). (D) Analysis of cell apoptosis in excised GDR tumors. Scale bar = 200 μm. (E) H&E staining of the paraffin-embedded sections of the GDR tumor tissues (scale bar = 250 μm).

The activity of empty ZIF-8@HA NPs in combination with gefitinib was also explored using the combination index (CI) originally described by Chou and Talalay 25. We obtained a CI value of 0.76±0.06, which indicates a synergistic effect of ZIF-8@G@HA. These observations further support the potentiation of gefitinib's therapeutic efficacy when incorporated into ZIF-8@G@HA NPs. More importantly, when GDR cells were treated with ZIF-8@G@HA, a significantly higher fraction of apoptotic cells was detected compared with cells that received either gefitinib or ZIF-8@HA (Fig. 4F, G). Consistent with the *in vitro* findings, GDR xenograft bearing mice treated with ZIF-8@G@HA were found to have a significantly reduced tumor growth, increased tumor cell apoptosis, and enhanced cystic space of necrosis when compared with mice treated with single agents (Fig. 5A-E). Although the ZIF-8@G@HA NPs exhibit similar efficacy compared to the combination treatment of ZIF-8 and gefitinib, it is important to note that the dose of gefitinib within the ZIF-8@G@HA NPs was only 0.6mg/kg, which is 42-fold lower than that used in the combination treatment group (25mg/kg). These findings suggest that ZIF-8@G@HA offers the advantages of targeted drug delivery, allowing for a more precise drug localization, and may enhance the therapeutic effects of gefitinib. The use of ZIF-8@G@HA NPs allows for a more effective delivery of gefitinib while minimizing the potential for systemic side effects. Collectively, these findings suggest that ZIF-8@G@HA NPs exhibit a promising therapeutic activity capable of surmounting gefitinib resistance using a two-in-one nano-system which simultaneously harbors a payload of gefitinib in the zinc nanoplatform.

### The overloading of Zn^2+^ is responsible for the tumor cell killing

**Figure 6:**
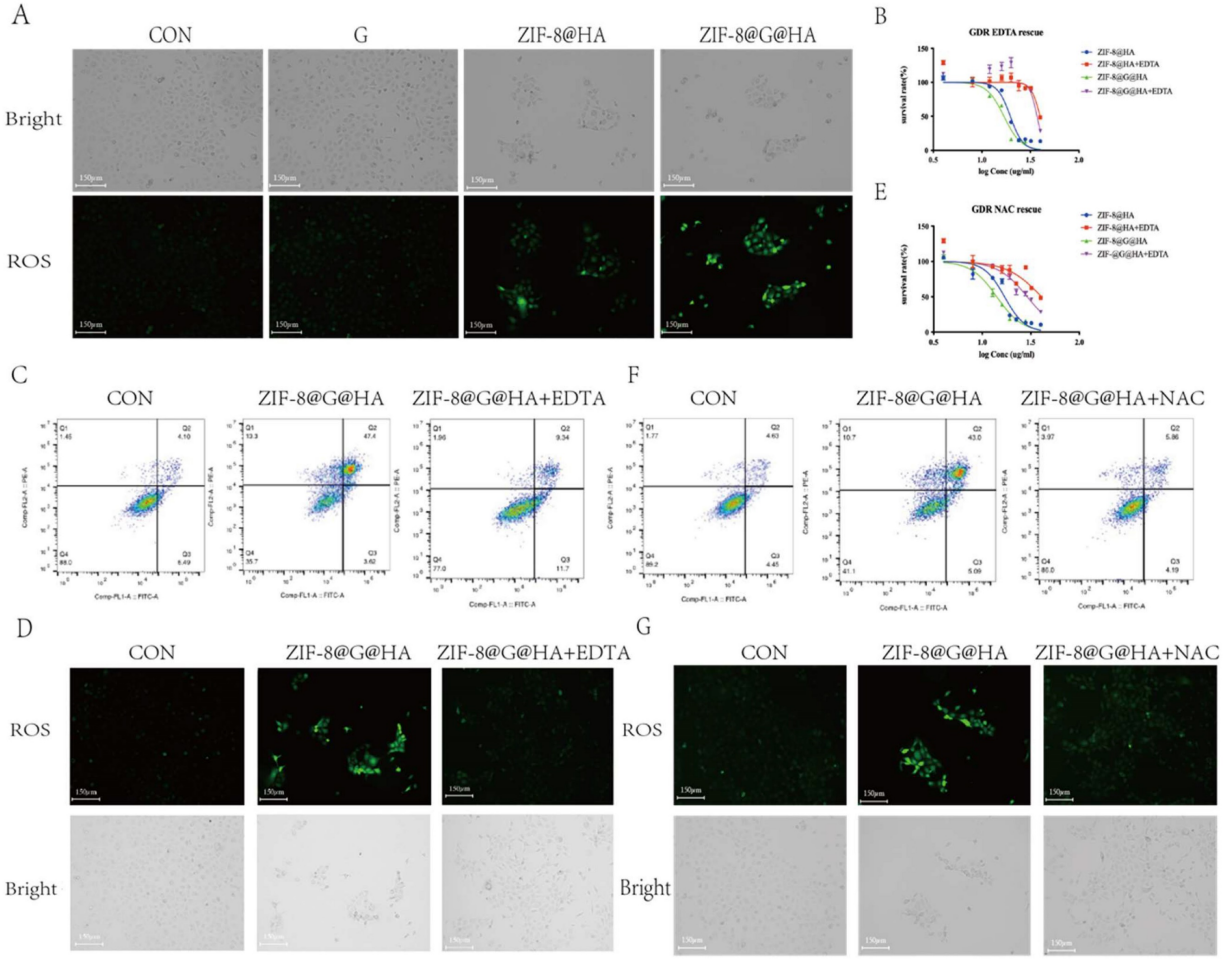
** Release of Zn^2+^ and generation of ROS mediating tumor cell killing.** (A) Detecting ROS production in HCC827 cells after treatments of gefitinib/ZIF-8@HA/ZIF-8@G@HA for 24h via DCFH-DA staining. Scale bar = 150 μm. (B) Determination of HCC827 cell viability after NPs treatments and EDTA rescue. (C) Cell apoptosis of HCC827 cells after NPs treatments and EDTA rescue. (D) Fluorescence microscopy images of ROS generation in HCC827 cells after NPs treatments and EDTA rescue. Scale bar = 150 μm. (E) Determination of HCC827 cell viability after NPs treatments and NAC rescue. (F) Cell apoptosis of HCC827 cells after NPs treatments and NAC rescue. (G) Fluorescence microscopy images of ROS generation in HCC827 cells after NPs treatments and NAC rescue (scale bar = 150 μm).

Although ROS are essential for cellular signaling, excessive ROS production drives mitochondrial dysfunction and leads to cell death 26, 27. Several studies revealed the cause-effect relationships between zinc accumulation and ROS generation 28, 29. To explore the mechanism underlying the overcoming of gefitinib resistance by ZIF-8@G@HA NPs, we studied ROS production in treated GDR cells via staining with 2,7-dichlorodihydrofluorescein diacetate (DCFH-DA), which is a fluorescent indicator of ROS. No ROS production was observed in GDR cells incubated with either PBS or gefitinib. In contrast, ROS was produced when either ZIF-8@HA or ZIF-8@G@HA were introduced to GDR cells with ZIF-8@G@HA treatment driving more ROS production than the empty NPs (Fig. 6A). Since the zinc capsule was shown to break down upon cellular uptake, we suspected that ROS production observed in GDR cells was driven by Zn^2+^ release in the cytoplasm upon intracellular breakdown of the Zn^2+^ nanoplatform.

To verify that the Zn^2+^ released from the Zn^2+^ nanoplatform is responsible for the reversal of gefitinib resistance via promotion of tumor cell death, we applied ethylenediaminetetraacetic acid (EDTA) to nanoparticle-treated GDR cells at a non-cytotoxic concentration of 342.2μM to chelate the Zn^2+^ ions released from the zinc nanoplatform. An MTT assay and flow cytometric analysis were performed using GDR cells after nanoparticle administration with/without EDTA to demonstrate the cytotoxic activity of Zn^2+^. The corresponding IC_50_ values of the different groups are depicted in the Table S1. The IC_50_ showed that the administration of EDTA significantly attenuated the Zn^2+^ nanoparticle-induced decrease in cell viability (Fig. 6B-C).

**Figure 7:**
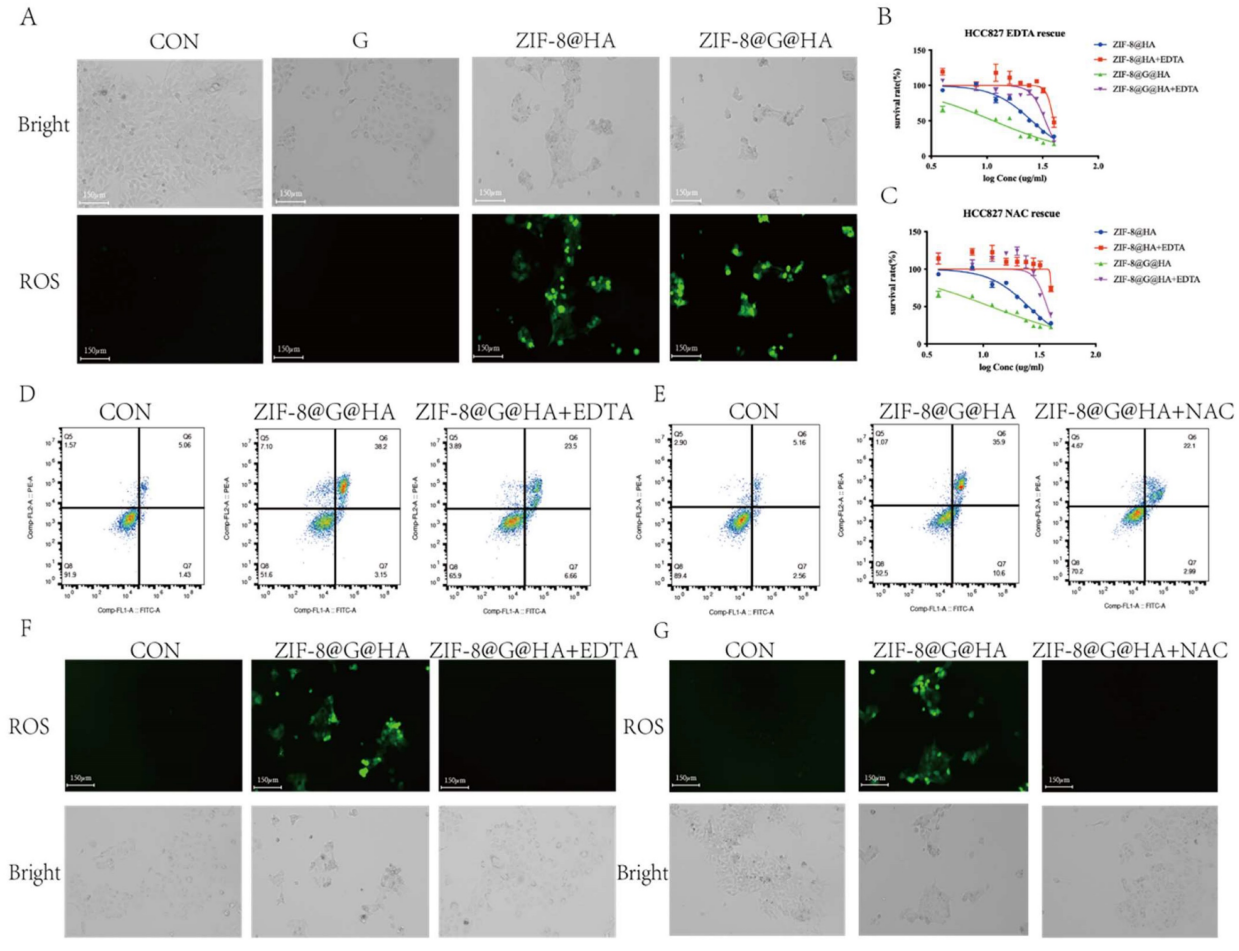
** Release of Zn^2+^ and generation of ROS medaiting the reversal of gefitinib resistance and tumor cell killing.** (A) Detection of ROS production in GDR cells after treatments of gefitinib/ZIF-8@HA/ZIF-8@G@HA for 24h and staining with DCFH-DA. Scale bar = 150 μm. (B) Determination of HCC827 cell viability after NPs treatments and EDTA rescue. (C) Determination of GDR cell viability after NPs treatments and NAC rescue. (D) Cell apoptosis of GDR cells after NPs treatments and EDTA rescue. (E) Cell apoptosis of GDR cells after NPs treatments and NAC rescue. (F) Fluorescence microscopy images of ROS generation in GDR cells after NPs treatments and EDTA rescue. Scale bar = 150 μm. (G) Microscope images of ROS generation in GDR cells after NPs treatments and NAC rescue (scale bar= 150 μm).

DCFH-DA staining was also performed; the results clearly illustrate that the Zn^2+^ chelation mediated by EDTA could impede ROS generation driven by the administration of ZIF-8@G@HA NPs (Fig. 6D). These results indicate that limiting the intracellular release of Zn^2+^ via chelating zinc ions with EDTA could attenuate the anti-tumor effects of the nanoparticles in GDR cells. To verify that the Zn^2+^-mediated ROS generation contributes to the reversal of gefitinib resistance via promotion of tumor cell death, we applied the antioxidant agent N-acetylcysteine (NAC) to scavenge ROS generated due to Zn^2+^ release. The MTT assay, Annexin-V/PI double-staining assay and representative fluorescence images demonstrated that NAC abrogated Zn^2+^-induced ROS-dependent cell damage and significantly attenuated the ZIF-8 NP-induced cell viability decrease (Fig. 6 E-G; Table S1). Taken together, the use of EDTA to chelate zinc ions and NAC to entrap ROS confirmed that Zn^2+^ and ROS play crucial roles in overcoming gefitinib resistance via promotion of tumor cell death in GDR cells. Moreover, our experiments demonstrate that treatment of parental cells with ZIF-8@HA NPs and ZIF-8@G@HA resulted in the generation of ROS as depicted in Fig. 7A. Importantly, when we used the combination of EDTA+NAC to chelate Zn^2+^ and entrap ROS, respectively, we significantly reduced available ROS levels (Fig. 7 F, G). However, we observed that even with the combined treatment of EDTA+NAC, ZIF-8@G@HA-induced cell death in parental HCC827 cells was only partially halted. This can be attributed to the presence of gefitinib embedded within the ZIF-8@G@HA NPs (Fig. 7B-E; Table S1). Collectively, these results indicate that the released Zn^2+^ cations and consequent ROS generation resulting from the breakdown of ZIF-8 NPs, are responsible for the direct tumor cell killing.

### The difference between parental HCC827 and GDR cells

**Figure 8:**
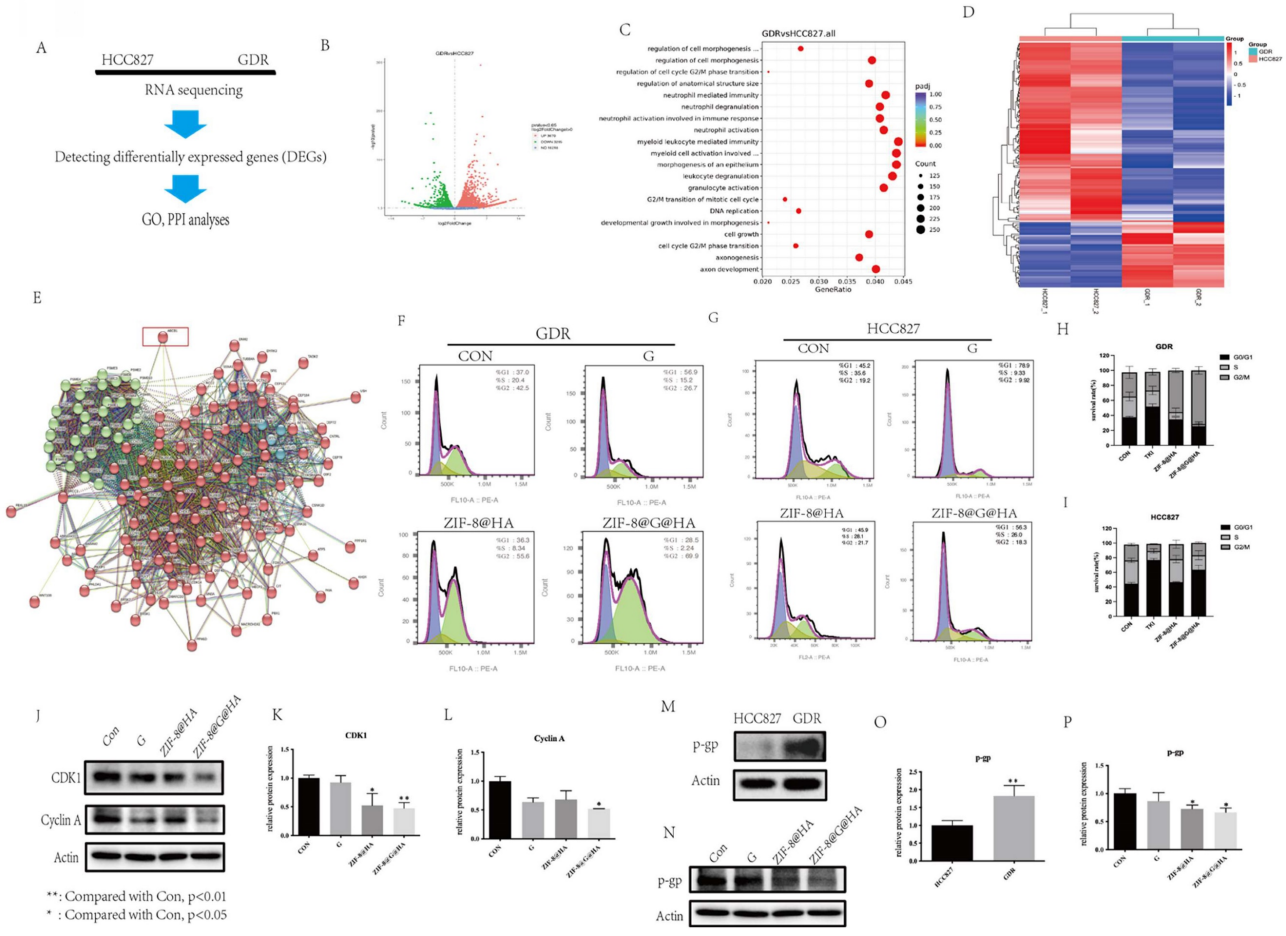
** The difference between parental HCC827 and GDR cells and the mechanism underlying the reversal of gefitinib resistance.** (A) The flow chart of RNA-sequencing analysis among parental HCC827 and GDR cells. (B) Volcano plot of differentially expressed genes between HCC827 and GDR cells (C) Gene ontology (GO) analysis among differentially expressed genes. (D) Heat map among cell cycle related genes in HCC827 and GDR cells. (E) Protein-protein interaction (PPI) analysis among cell cycle related genes. (F,H) Cell cycle phase analysis in GDR cells after treatments of gefitinib/ZIF-8@HA/ZIF-8@G@HA for 24h. (G,I) Cell cycle phase analysis in HCC827 cells after treatments of gefitinib/ZIF-8@HA/ZIF-8@G@HA for 24h. (J-L) Expression levels of cell cycle related markers in GDR cells after treatments of gefitinib/ZIF-8@HA/ZIF-8@G@HA for 24h. (M,O) Expression levels of P-gp among HCC827 cells and GDR cells. (N,P) Expression of P-gp in GDR cells after treatments of gefitinib/ZIF-8@HA/ZIF-8@G@HA for 24h.

To gain insights into the differential sensitivity of GDR cells to the ZIF-8@HA nanoparticles, we performed RNA sequencing on both parental HCC827 and GDR cells (Fig. 8A). The results of the volcano plots revealed that of the 6965 differentially expressed genes (DEGs), 3670 were upregulated and 3295 were downregulated in GDR cells as compared to parental cells (Fig. 8B). Gene ontology and protein-protein interaction (GO and PPI, respectively) analyses were further conducted using the RNA sequencing data. The GO analysis was first conducted for the DEGs; there are up to one thousand gene functions which were significantly altered, we therefore listed the top 20 significantly enriched functions. We found that those functions were mainly related to G2/M cell cycle progression and cell morphogenesis (Fig. 8C). We selected those significant cell cycle-related genes that were changed and further conducted hot mapping and PPI analyses. As evident from the hot mapping, the levels of cell cycle-related genes in GDR cells were obviously distinct from parental cells (Fig. 8D). The PPI results revealed that these cell cycle-related genes could mainly be divided into two clusters, and the ABCB1 gene encoding for P-gp, appears to have connections with both clusters (Fig. 8E). P-gp, a major multidrug efflux transporter has been reported to induce multidrug resistance in cancer cells via an ATP-dependent extrusion of multiple structurally and functionally distinct anticancer drugs out of cancer cells 30-33. Thus, we hypothesized that P-gp might partake in cell cycle progression and our NPs could reverse gefitinib resistance via regulating cell cycle and P-gp expression levels. To provide supportive evidence for this hypothesis, the impact of NPs on cell cycle progression was also analyzed using flow cytometry. As evident from Fig. 8F,8H, the cell cycle was found to be arrested at the G2/M phase in GDR cells after nanoparticle treatment, and an enhanced effect could be observed after treatment with ZIF-8@G@HA. For parental cells, the single treatment with ZIF-8@HA NPs had no significant effect on cell cycle progression at the same concentration (Fig. 8G,I). This suggests that the therapeutic activity of ZIF-8@HA NPs may be more pronounced in GDR cells compared to parental cells. This finding was also consistent with the reduction in the protein levels of G2/M related cell cycle markers, CDK1 and cyclin A, in GDR cells treated with the nanoparticles, especially upon ZIF-8@G@HA treatment (Fig. 8J-L). Furthermore, we examined the protein expression level of P-gp in parental and GDR cells. P-gp was found to be upregulated in GDR cells, suggesting the contribution of P-gp to gefitinib resistance in GDR cells (Fig. 8M,O). Thus, we next tested the effects of the nanoparticles on P-gp protein levels. For GDR cells that underwent ZIF-8@HA/ZIF-8@G@HA treatment, P-gp protein levels were found to be lower when compared with GDR cells that were treated with gefitinib (Fig. 8N,P). These results indicate that Zn^2+^ could reverse gefitinib resistance in GDR cells through inhibition of P-gp expression, thereby inducing cell cycle arrest at the G2/M phase. Hence, encapsulation of gefitinib in zinc nanoparticles could enhance the cell cycle arrest effect.

## Discussion

The discovery of EGFR activating mutations in select lung cancer patients has led to the introduction of EGFR-TKIs for the treatment of NSCLC, particularly in patients harboring these EGFR mutations. EGFR-TKIs have demonstrated remarkable improvements in the clinical outcomes of these patients. However, although initially effective, acquired resistance to EGFR-TKIs typically arises within a year due to the emergence of clonal variants harboring multiple mechanisms of chemoresistance 34. Hence, there is a pressing need to develop efficacious therapeutic approaches capable of overcoming acquired EGFR-TKI resistance in these patients. Currently, two primary strategies exist to address this acquired chemoresistance. The first approach involves the utilization of next-generation EGFR-TKIs in lieu of first-generation EGFR-TKIs. This strategy proved particularly effective in cases where patients exhibit EGFR mutations such as T790M, which are associated with the development of TKI resistance 35. Another strategy employed to surmount acquired resistance involves the concurrent administration of diverse classes of therapeutic agents along with EGFR-TKIs, such as the combination of mesenchymal-epithelial transition (MET) receptor inhibitors and EGFR-TKIs 4, 36. Specifically, EGFR-TKIs which were combined with monoclonal antibodies targeting the MET receptor tyrosine kinase, have been studied in patients with advanced NSCLC with deregulated *MET*, predominantly due to exon 14 skipping mutations or *MET* gene amplification. In this respect, some MET inhibitors including capmatinib and tepotinib, have proven to be highly effective in this molecularly defined subgroup of NSCLC patients and have been already approved for clinical use 37. Furthermore, the combination of EGFR-TKIs with immune checkpoint inhibitors (ICIs) has also been proposed 38-40.

Zn^2+^ is an absolutely essential micronutrient that plays a key role in the structural or enzymatic functions of many cellular proteins 41. However, excessive intracellular levels of Zn^2+^ can have irreversible toxic effects on cells, triggering the generation of ROS, also leading to impaired ATP production. Zn^2+^ is emerging as an important signaling divalent cation in the development and progression of cancer 42. Notably, tumor cells are more vulnerable to Zn^2+^ accumulation compared to normal cells due to differences in Zn^2+^ metabolism and homeostasis pathways 8, 42. Specifically, since most intracellular zinc is either compartmentalized or associated with proteins, free intracellular zinc levels are at the picomolar to low nanomolar range 43. Therefore, the potential of utilizing Zn^2+^-mediated ROS generation to enhance the therapeutic efficacy of gefitinib and overcome gefitinib resistance is of great interest. In this respect, to date, no specific studies have demonstrated the effectiveness of this Zn^2+^-based strategy to enhance the efficacy of gefitinib treatment and overcome gefitinib resistance.

The current study had two primary objectives: firstly, to investigate the potential of combining Zn^2+^ with gefitinib to enhance the efficacy of gefitinib in lung cancer cells and possibly overcome acquired drug resistance. Secondly, it will streamline the administration modality and enhance efficacy by devising and optimizing a two-in-one nanotherapeutic system. To achieve these goals, we developed a simple, biocompatible, and effective nano-interrupter termed ZIF-8@G@HA, which incorporates Zn^2+^. Our findings revealed that ZIF-8@G@HA not only enhanced the efficacy of gefitinib in parental HCC827 cells but also exhibited superior activity against GDR cells. Consistently, the CI values in both the HCC827 and GDR cell lines were found to be substantially less than 1, indicating a significant synergy between the release of Zn^2+^ and gefitinib. Furthermore, our *in vitro* and *in vivo* studies provide further evidence of the synergistic activity achieved through this combined treatment using Zn^2+^ release and other therapeutic agents in both parental and GDR cells. In summary, our study demonstrated the potential of combining Zn^2+^ and gefitinib, utilizing the ZIF-8@G@HA nano-interrupter, to overcome gefitinib resistance and enhance preclinical therapeutic outcomes in lung cancer *in vivo*.

To gain deeper insights into the anti-tumor and the mechanism underlying the apparent surmounting of drug resistance by ZIF-8@G@HA, we investigated ROS generation after various treatments. Interestingly, we observed a significant increase in intracellular ROS levels following NPs treatment, while gefitinib alone had no notable effect on ROS generation. Moreover, we conducted additional experiments to eliminate the impact of Zn^2+^ and Zn^2+^-induced ROS using EDTA and ROS scavenging, respectively. The results revealed a significant protection from the cytotoxic activity of ZIF-8@G@HA, emphasizing the critical role of Zn^2+^ in tumor cell killing and the overcoming of gefitinib resistance.

It is noteworthy that GDR cells were found to be more sensitive to the release of Zn^2+^ compared to their parental cells. This implies that GDR cells may have specific molecular or genetic characteristics that render them more susceptible to the cytotoxic activity of Zn^2+^. Deciphering the molecular mechanisms underlying this differential sensitivity could potentially provide valuable insights into the development of targeted therapies for the overcoming of gefitinib resistance in cancer. To investigate the underlying basis of this increased sensitivity of GDR cells to Zn^2+^, we conducted RNA sequencing in GDR and parental cells. This analysis revealed that GDR cells exhibited high P-gp expression, a dominant multidrug efflux transporter known to mediate cancer MDR. It has been reported that excessive intracellular Zn^2+^ levels can influence cell cycle progression 44. Building upon these findings, we observed that under the same concentration, the NPs induced a G2/M arrest exclusively in GDR cells but not in their parental cells. Furthermore, treatment with ZIF-8@G@HA significantly reduced P-gp expression levels in GDR cells.

Collectively, our results suggest that ZIF-8@G@HA induces ROS generation, causes cell cycle arrest, and promotes the accumulation of gefitinib in tumor cells, also by impairing P-gp function. Consequently, this leads to the reversal of gefitinib resistance in GDR cells. The combined activity of ROS generation, cell cycle arrest, and enhanced gefitinib accumulation contribute to the enhanced efficacy of ZIF-8@G@HA in overcoming gefitinib resistance.

## Conclusion

The current construction of the ZIF-8@G@HA nano-interrupter offers a promising therapeutic approach to enhance the efficacy of gefitinib in HCC827 cells and to overcome gefitinib resistance. This study introduces a simple, biocompatible, and effective strategy that holds significant promise for future clinical applications in the treatment of lung cancer.

## Supplementary Material

Supplementary table.

## Figures and Tables

**Schematic 1:**
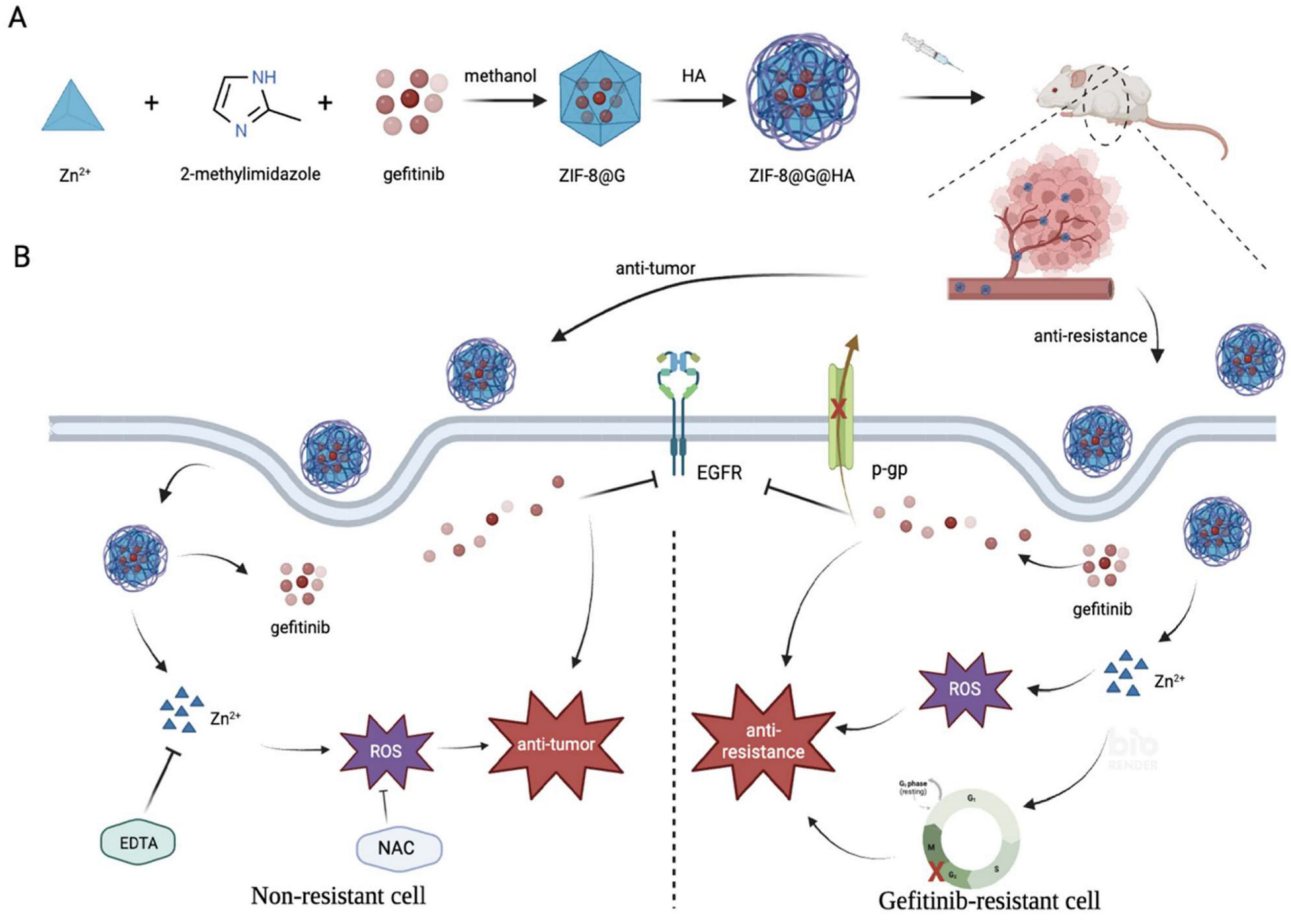
a) Preparation procedure of zeolitic imdazolate framework-8@Gefitinib@hyaluraonic nanoparticle (ZIF-8@G@HA nano-interrupters). b) Schematic illustration for tumor killing and overcoming gefitinib resistance by ZIF-8@G@HA nano-interrupters.
