# Supplementary material for: Nanoenabled intracellular zinc bursting for efficacious reversal of gefitinib resistance in lung cancer
Source: Int J Biol Sci. 2024 May 19;20(8):3028–45. doi: 10.7150/ijbs.95929 (PMC11186351; doi:10.7150/ijbs.95929)
Supplement: Supplementary file 1 — Supplementary table. [file ijbsv20p3028s1.pdf]

Supplementary Data

**GDR**	ZIF-8@HA	ZIF-8@HA+EDTA	ZIF-8@G@HA	ZIF-8@G@HA+EDTA
**IC_{50} (ug/ml)**	15.65±0.70	39.8±3.39	10.89±0.53	37.02±3.25
**GDR**	ZIF-8@HA	ZIF-8@HA+EDTA	ZIF-8@G@HA	ZIF-8@G@HA+EDTA
**IC_{50} (ug/ml)**	15.98±0.52	40.87±0.60	10.11±0.29	35.05±1.29
**HCC827**	ZIF-8@HA	ZIF-8@HA+EDTA	ZIF-8@G@HA	ZIF-8@G@HA+EDTA
**IC_{50} (ug/ml)**	25.26±0.17	40.52±5.42	11.67±0.67	36.99±1.95
**HCC827**	ZIF-8@HA	ZIF-8@HA+EDTA	ZIF-8@G@HA	ZIF-8@G@HA+EDTA
**IC_{50} (ug/ml)**	25.26±0.17	39.73±5.8	11.4±0.12	33.48±0.89

Table S1. The IC_{50} of NPs treatments with/without EDTA and NAC in HCC827/GDR cells.
